# Multisystemic fusariosis with fulminant evolution^☆^^☆☆^

**DOI:** 10.1016/j.abd.2020.03.007

**Published:** 2020-07-12

**Authors:** Nelson Turra, Agustina Acosta, Andrea Incoronato, Pilar Beltramo

**Affiliations:** a Department of Dermatology, Dr. Manuel Quintela Clinical Hospital, Montevideo, Uruguay; b Department of Pediatric Dermatology, Pereira Rossell Hospital, Montevideo, Uruguay; c Department of Hemato-oncology, Pereira Rossell Hospital, Montevideo, Uruguay; d Department of Pathology, Pereira Rossell Hospital, Montevideo, Uruguay

**Keywords:** Bacterial infections and mycoses, Bone marrow transplantation, Central nervous system fungal infections, Fusariosis, Fusarium

## Abstract

This report presents the case of a 13-year-old female patient with history of acute myeloid leukemia, who, after a bone marrow transplant, began to vomit and experienced rapidly progressive deterioration of consciousness, in addition to disseminated erythematous-violaceous macules, and some blisters with hemorrhagic content inside. Skin biopsy evidenced intravascular filamentous structures. A blood culture confirmed the presence of *Fusarium oxysporum*. Intravenous treatment with voriconazole was initiated. The patient evolved unfavorably with multiple necrotic skin lesions, ischemic brain lesions, and death.

## Introduction

*Fusarium* spp. are fungi universally found in soil, air, and plants; they mainly affect immunocompromised patients and may cause localized, focally invasive, or disseminated disease.^1^, ^2^ Immunosuppressed patients and carriers of hematologic malignancies are very susceptible to the development of invasive forms, showing high mortality.^2^, ^3^, ^4^ This report describes the case of a patient with acute myeloid leukemia, who presented with invasive fusariosis with fulminant evolution after a bone marrow transplant.

## Case report

13-year-old adolescent, female, who presented three years ago with acute myeloblastic leukemia, achieving complete remission of her disease. She presented her first hematological relapse three months ago, then achieved a new complete remission, and under these conditions an allogeneic bone marrow transplant (BMT) was planned. Myelosuppressive conditioning was performed with busulfan and fludarabine, administered together with an infectious prophylaxis with acyclovir, trimethoprim-sulfamethoxazole, and fluconazole. After five days of the transplant, administration of granulocyte colony-stimulating factor was initiated (Neupogen® 300 µg/kg/day).

**Figure 1:**
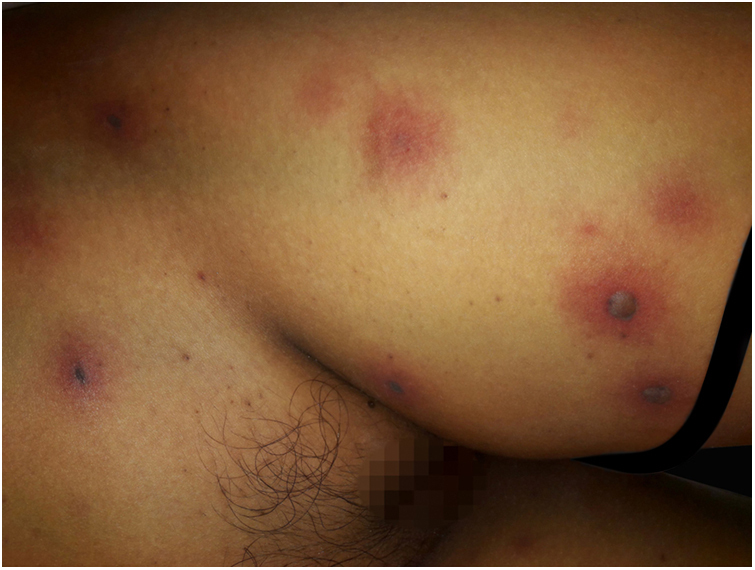
Invasive fusariosis, early clinical manifestations (left thigh). Dermatosis characterized by the presence of erythematous-violaceous macules, which coexist together with hemorrhagic blisters, situated on erythematous and infiltrated skin.

After a week of BMT, the patient began to experience headache and vomiting, showing central facial paralysis and rapidly progressive deterioration of consciousness, until reaching a score of 7 according to the Glasgow Scale; this motivated her admission to the Intensive Care Unit. The antibiotic treatment was adjusted to a broad spectrum scheme with meropenem 60 mg/kg/day and vancomycin 40 mg/kg/day. At that time, an interconsultation with a dermatologist was performed, due to the sudden appearance of multiple disseminated skin lesions. On physical examination, she presented disseminated dermatosis on the trunk, lower, and upper limbs, characterized by rounded erythematous-violaceous macules, infiltrated on palpation, and some blisters with hemorrhagic content inside (Fig. 1). Laboratory tests showed hemoglobin of 9.4 mg/dL; 2,800 mm^3^ leukocytes (60% neutrophils); 53,000 mm^3^ platelets, with normal liver and kidney function. Blood cultures were negative. Also, polymerase chain reaction was negative for cytomegalovirus, varicella zoster virus, and herpes simplex virus types 1 and 2.

**Figure 2:**
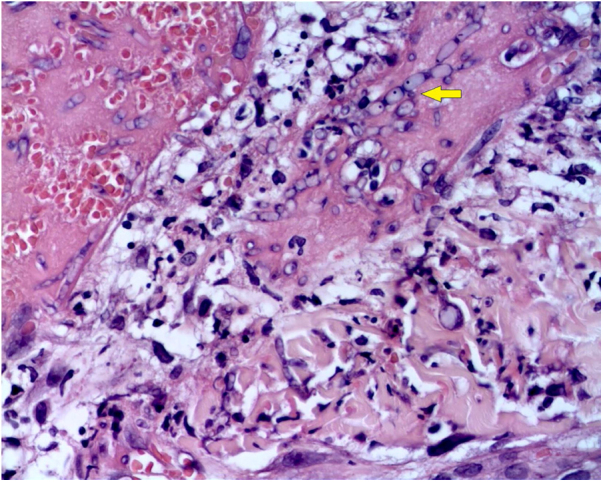
Fusariosis, histopathology of skin lesions (Hematoxylin & eosin, ×40). Vessels with fibrinoid wall necrosis and occlusive thrombosis in their lumens, accompanied by a superficial perivascular inflammatory infiltrate composed of lymphocytes, neutrophils, and leukocytoclasia. The yellow arrow highlights the presence of intravascular filamentous structures, corresponding to septate hyphae.

**Figure 3:**
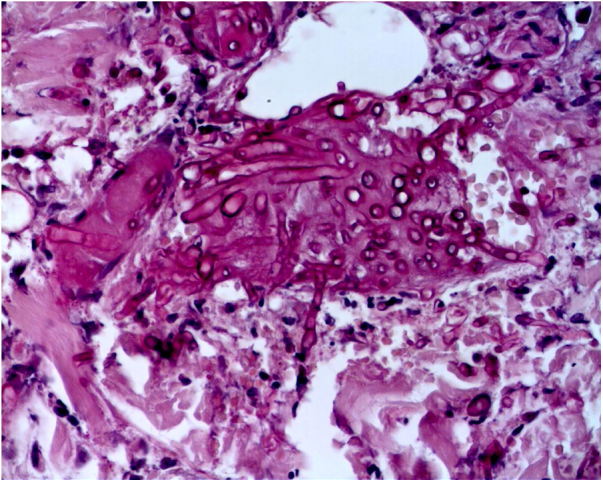
Fusariosis, histopathology of skin lesions (PAS, ×40). Septate hyphae and intravascular conidiophores are evident.

**Figure 4:**
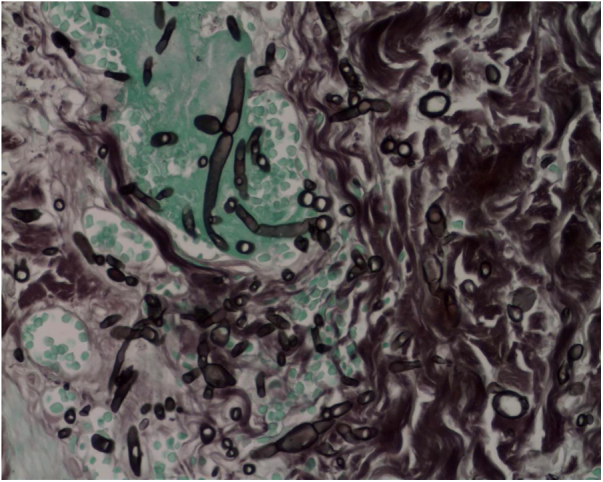
Fusariosis, histopathology of skin lesions (Grocott-Gomori, ×40). Septate hyphae and intravascular conidiophores are evident.

**Figure 5:**
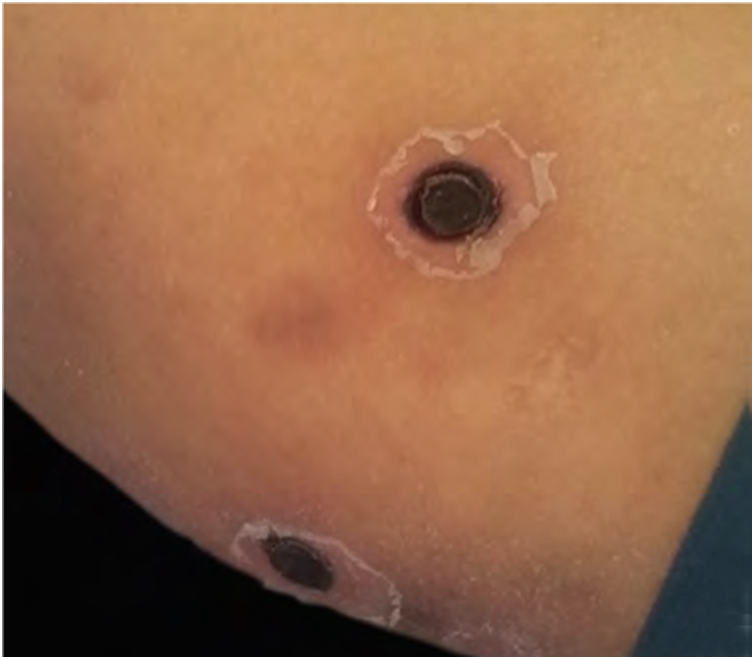
Invasive fusariosis, late clinical manifestations (right arm). Dermatosis characterized by the presence of necrotic skin lesions with scaling collarette.

The skin biopsy was processed for staining with hematoxylin-eosin, PAS, and Grocott-Gomori; evidencing intraepidermal acantholysis, vessels with fibrinoid necrosis of the wall, and occlusive thrombosis. The biopsy also found superficial perivascular inflammatory infiltrate composed of lymphocytes, neutrophils, and leukocytoclasia, highlighting the presence of intravascular filamentous structures, corresponding to septate hyphae and conidiospores (Figure 2, Figure 3, Figure 4). A subsequent blood culture confirmed the presence of *Fusarium oxysporum*, and treatment was started with intravenous voriconazole 4 mg/kg/dose, twice a day. The patient evolved unfavorably and with skin lesions that adopted a central necrotic appearance with a scaling collarette (Fig. 5). Subsequently, she began to suffer from severe diabetes insipidus (hypernatremia crisis of up to 170 mEq/L). Magnetic resonance of the brain, taken 20 days post-transplant, evidenced the presence of extensive ischemic lesions and cerebral edema, with tonsillar herniation and bulbo-spinal compression. After three weeks of the transplant, the patient died.

## Discussion

*Fusarium* spp. are fungi present in the soil in all parts of the world, including tropical, desert, and even arctic regions; this is due to their high adaptability. Regarding their incidence, they corresponds to the second most frequent cause of invasive mold infection in hemato-oncological patients, behind Aspergillus spp.^5^ The risk factors for invasive fusariosis are severe neutropenia, poor cellular immunity, recent induction chemotherapy for leukemia, recent BMT, and graft vs. host disease.^1^, ^6^ Likewise, the authors highlight that the current patient was a carrier of acute myeloid leukemia and had recently received a BMT. It is expected that after a BMT, severe neutropenia will occur, in the context of the myelosuppressive conditioning required for this procedure.

Invasive forms of fusariosis show cutaneous involvement in 63% of cases, and this is characterized by the presence of subcutaneous nodules, erythematous macules, or vesicle-pustules that may become necrotic.^4^, ^7^ This pathogen is highly angio-invasive, producing tissue infarctions such as those evidenced in the brain of the current patient, which generate intravascular sporulation, favoring a greater sensitivity of blood cultures in the final stages of the disease.^8^ *Fusarium* spp. are resistant to multiple antifungals, but are highly susceptible to amphotericin B and voriconazole.^1^, ^9^, ^10^ However, severe neutropenia is the most important prognostic factor, determining lethality of up to 75% of cases.^6^, ^10^

## Final considerations

Invasive fusariosis is extremely serious and is associated with high mortality in immunosuppressed patients. It requires intensive antifungal treatment and administration of granulocyte colony stimulating factor, since its prognosis is closely related to the recovery of neutropenia. The role of the dermatologist as part of a multidisciplinary team must be emphasized, having a high diagnostic suspicion of this pathology in the case of immunosuppressed patients with clinical manifestations similar to those previously elucidated.

## Financial support

None declared.

Authors' contributions

Nelson Turra: Approval of the final version of the manuscript; drafting and editing of the manuscript; participation in study design; participation in the therapeutic and/or propaedeutic conduct of the studied cases; critical review of the literature; critical review of the manuscript.

Agustina Acosta: Approval of the final version of the manuscript; participation in study design; participation in the therapeutic and/or propaedeutic conduct of the studied cases; critical review of the literature; critical review of the manuscript.

Andrea Incoronato: Approval of the final version of the manuscript; participation in study design; participation in the therapeutic and/or propaedeutic conduct of the studied cases; critical review of the literature; critical review of the manuscript.

Pilar Beltramo: Approval of the final version of the manuscript; participation in study design; participation in the therapeutic and/or propaedeutic conduct of the studied cases; critical review of the literature; critical review of the manuscript.

## Conflicts of interest

None declared.
